# How Green is Your Plasticizer?

**DOI:** 10.3390/polym10080834

**Published:** 2018-07-28

**Authors:** Roya Jamarani, Hanno C. Erythropel, James A. Nicell, Richard L. Leask, Milan Marić

**Affiliations:** 1 Department of Chemical Engineering, McGill University, 3610 University St, Montréal, QC H3A 0C5, Canada; roya.jamarani@mail.mcgill.ca (R.J.); hanno.erythropel@yale.edu (H.C.E.); richard.leask@mcgill.ca (R.L.L.); 2 Center for Green Chemistry and Green Engineering, Yale University, 370 Prospect St, New Haven, CT 06511, USA; 3 Department of Civil Engineering & Applied Mechanics, McGill University, 817 Sherbrooke Street West, Montreal, QC H3A 0C3, Canada; jim.nicell@mcgill.ca

**Keywords:** additive, plasticizer, phthalate, toxicity, biodegradation, leaching, metabolites

## Abstract

Plasticizers are additives that are used to impart flexibility to polymer blends and improve their processability. Plasticizers are typically not covalently bound to the polymers, allowing them to leach out over time, which results in human exposure and environmental contamination. Phthalates, in particular, have been the subject of increasing concern due to their established ubiquity in the environment and their suspected negative health effects, including endocrine disrupting and anti-androgenic effects. As there is mounting pressure to find safe replacement compounds, this review addresses the design and experimental elements that should be considered in order for a new or existing plasticizer to be considered green. Specifically, a multi-disciplinary and holistic approach should be taken which includes toxicity testing (both in vitro and in vivo), biodegradation testing (with attention to metabolites), as well as leaching studies. Special consideration should also be given to the design stages of producing a new molecule and the synthetic and scale-up processes should also be optimized. Only by taking a multi-faceted approach can a plasticizer be considered truly green.

## 1. Introduction

Plasticizers are additives, typically small organic molecules, that decrease the glass transition temperature (*T*_g_) of the polymer they are blended with, creating flexible or semi-rigid products with improved processing characteristics [1]. Approximately 90% of all globally produced plasticizers are used to make flexible poly(vinyl chloride) (PVC), with di(2-ethylhexyl) phthalate (DEHP) being the most frequently used plasticizer [2]. Plasticizers can be classified as either internal or external. Internal plasticizers achieve flexibility by lowering *T*_g_ through grafting or copolymerization of softer monomer units to the polymer chain, while external plasticizers, such as DEHP, are simply blended with the polymer at elevated temperatures and do not form covalent bonds [3]. Internal plasticizers are less commonly used, often for specific purposes, because the fixed chemical bonds offer less freedom and limited properties compared to external plasticizers. External plasticizers offer higher flexibility to adjust the final polymer properties, given that the plasticizer is added after polymerization [1,3]. Additionally, the type and amount of plasticizer can be carefully tailored to produce a wide variety of formulations and product properties and impart different levels of flexibility depending on the desired application. Furthermore, because no chemical reaction is involved, external plasticization also tends to be more cost-effective, and are thus used to a greater extent. Therefore, this review will focus exclusively on external plasticizers.

The lack of a chemical bond between external plasticizers and polymers allows the plasticizer to diffuse within and out of the blend over time. Once plasticizer molecules reach the surface of the blend, leaching into their surroundings occurs that results in human exposure and entry of the compounds into the environment [4,5]. For example, DEHP and its metabolites have been found to be ubiquitous environmental contaminants, likely due to their slow degradation rates combined with high rates of entry into the environment [6,7]. Phthalate plasticizers including DEHP have been detected in a wide variety of environmental samples, including house dust [8,9,10], air [11], soil [12], watersheds [4], and animals [6]. This is especially problematic given that many studies have linked DEHP and its metabolite, mono(2-ethylhexyl) phthalate (MEHP), to endocrine disruption in human and animal models, and negative effects on male reproductive development (anti-androgenic effects) [13,14,15,16,17]. As a result of these findings, the use of DEHP and other phthalates has been regulated in consumer items such as children’s toys in many countries, including Canada [18], the United States [19], the European Union [20], and Japan [21]. Therefore, there is a need to develop alternative, safer, plasticizers.

**Figure 1:**
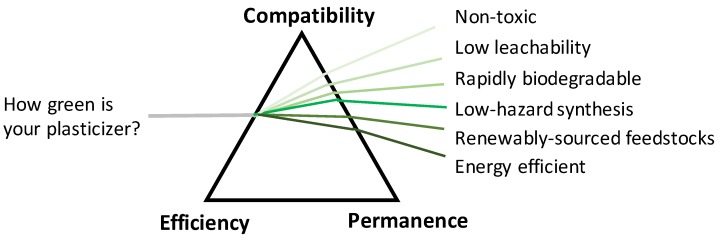
Green plasticizer design considerations. Adapted from R.F. Boyer, 1951 [22].

The traditional view of plasticizers has held that in order to develop a well-functioning plasticizer, a balance must be struck between the compatibility, efficiency, and permanence of the plasticizer blended with PVC, as reflected by the three vertices of the triangle pictured in Figure 1 [22]. This scheme reflects the fact that achieving desirable effects with respect to one of the properties can negatively impact upon other properties. For example, molecular features such as polar groups on a plasticizer are attracted to polar sites on the PVC molecule and will render the plasticizer more compatible with PVC; however, if only polar components are present in a plasticizer, its plasticizing effectiveness is not very high. Conversely, the non-polar segments of the molecule generally provide good plasticization, but if they are too large or numerous, the plasticizer might be poorly miscible with PVC and lead to exudation. This careful balancing act of optimizing plasticizer performance has been the primary focus of research and development work for many years. However, given the significant negative impacts of phthalate plasticizers noted above, in this review we endeavor to outline approaches to plasticizer design and evaluation that also incorporate elements of green chemistry thinking, in addition to traditional performance considerations [23,24]. Therefore, the schematic shown in Figure 1 reflects the inherent and growing importance of maintaining plasticizer performance while considering green design elements such as toxicity, biodegradation, and leaching in developing safe and effective plasticizers. In order to answer the question “how green is your plasticizer?”, we need to not only ensure that compounds meet the balanced criteria of an effective plasticizer, but we must assess the effects of the plasticizer from non-traditional measures, informed by green chemistry, shown in Figure 1.

**Figure 2:**
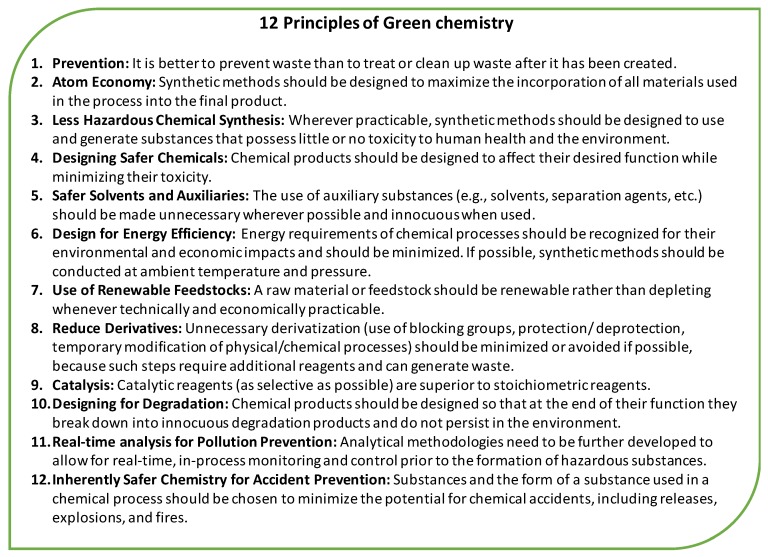
The 12 Principles of Green Chemistry. Anastas, P. T.; Warner, J. C. Green Chemistry: Theory and Practice, Oxford University Press: New York, NY, USA, 1998; p. 30. By permission of Oxford University Press [23].

At its core, the growing field of green chemistry aims to reduce or eliminate the use or generation of hazardous substances. This applies not only during the usage stage of a material, but also includes its production and end-of-life stages. A crucial component of green chemistry is thus the design stage, during which much of the future fate of a molecule or substance is already decided. Anastas and Warner first introduced the 12 principles of green chemistry, listed in Figure 2, and outlined the concept of green chemistry as a mindset in 1998 [23]. In brief, these principles offer guidance on how to design or improve materials and processes while adhering to the ideals of green chemistry. These principles include designing for degradation, designing benign or less toxic compounds, and preventing the production of waste, amongst others. A number of the remaining principles apply more specifically to the chemical synthesis itself such as using renewable feedstocks, using benign solvents, and improving atom economy [23]. To develop a truly green plasticizer, we propose to use these principles as a framework for design and testing.

Applying a holistic, multi-disciplinary approach that incorporates many of the green chemistry principles is essential for designing safe plasticizers. In order to do so, collaboration between chemists, toxicologists, biologists, and engineers, amongst others, is required. Unfortunately, however, research and development activities often focus on addressing a limited subset of the 12 principles (e.g., reducing human toxicity or designing for biodegradation), and are often undertaken with little input from other disciplines, and a compound will questionably be touted as green according to its performance with respect to those few selected criteria. In this paper we seek to outline the variety of green chemistry considerations that can apply to plasticizer design, highlighting those that are most important, and showing that plasticizer performance should be evaluated with respect to all of these relevant considerations in order to be considered green. That is, in addition to performing well as a functional plasticizer [25,26,27] a green plasticizer also needs to be (1) non-toxic and harmless to humans, animals, and the environment, (2) biodegrade quickly, without producing stable or toxic metabolites, and (3) leach as little as possible from the PVC blend. This review will focus mainly on these three principles since much of the experimental testing of new plasticizers will center upon them. Beyond these criteria, several other parameters, mostly pertaining to the synthesis of the compounds, such as using renewable feedstocks, maximizing atom economy, using safer solvents and reaction conditions, and using catalysts should also be considered. Additionally, life-cycle assessment (LCA) is another tool that can be used to assess the environmental impact of introducing a new compound to market [28]. In this review, we aim to demonstrate how to avoid toxicity, ensure biodegradability, and impart low leaching to plasticizers, while keeping all the different principles of green chemistry in mind.

## 2. Historical Perspective

PVC was first synthesized in the 1800s, but due to its poor processability in the absence of plasticizers and heat stabilizers it was not commercialized at that time [29]. It wasn’t until the early 20th century that the German chemist Fritz Klatte at Griesheim-Elektron started blending this hard and brittle polymer with esters and oils as ‘softeners’ that PVC could be produced commercially [3]. Thus, the idea of using plasticizers as key components of plastic formulations was born, allowing for the easy processing of PVC and its use in many different and diverse applications. By 1943, the demand for PVC products had increased considerably and there were already over 150 commercial plasticizers in use [30].

**Figure 3:**
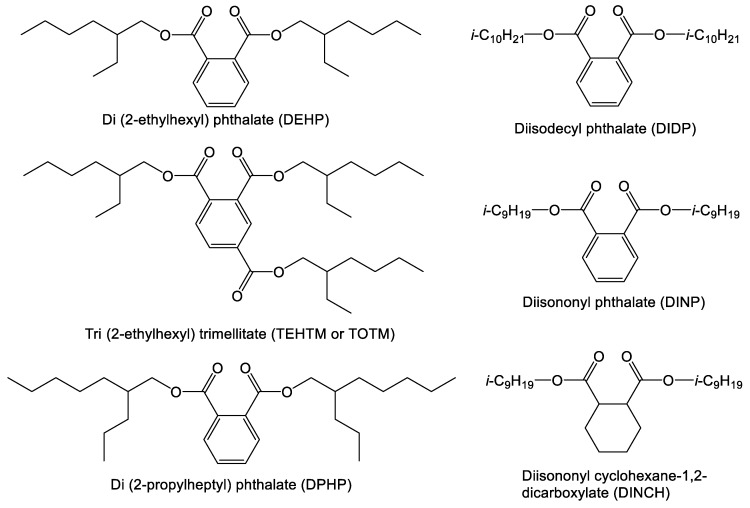
Chemical structures of four commercial phthalate plasticizers and two structurally similar compounds [39].

Esters of phthalic acid quickly became the most important class of plasticizers, and still remain so, due to their all-round plasticizing efficiency and low cost of production [3]. In particular, the compound DEHP (Figure 3) became the most widely used plasticizer [3,31]. Plasticized PVC products were increasingly manufactured due to their low cost, ease of fabrication, suitable mechanical properties, and compatibility with blood and medical solutions [32]. However, it was not until the 1980s that concerns over the deleterious health effects of plasticizers, such as DEHP, started to be more thoroughly investigated and the need for green replacement compounds was established [32,33,34]. The development of green consumer products is governed by conventional considerations such as cost reduction and performance enhancement that undoubtedly remain relevant to manufacturers but also by factors such as government regulation and spending, pressure from non-profit organizations and industry leaders, and consumer social awareness. These forces have become increasingly important in driving a more proactive and green approach to replacing problematic compounds.

The first step on the road to developing green plasticizers was the research that established the toxicity of DEHP and its metabolites [13,14,16]. This was followed by many studies on exposure that demonstrated the ubiquity of phthalate plasticizers in the environment and led to regulations requiring the labelling or banning of DEHP in various products [4,9,10,11,12,18,19,20,21]. In response to existing and looming regulations, a number of replacement compounds were introduced to the market [35]. Non-phthalate compounds, such as BASF’s Hexamoll DINCH^®^, Dow ECOLIBRIUM^TM^ and HallStar Hallgreen, were released commercially, amongst others [36,37]. Data from the European PVC industry [38] suggests that DEHP was mainly replaced by other phthalate plasticizers such as di(isononyl phthalate) (DINP), di(2-propylheptyl) phthalate (DPHP), and diisodecyl phthalate (DIDP), or structurally similar compounds such as trioctyl trimellitate (TOTM)–which is essentially DEHP with an added 2-ethylhexyl ester arm, and diisononyl cyclohexane 1,2-dicarboxylate (DINCH), which is hydrogenated DINP (see Figure 3) [38].

Still, data gaps exist in the evaluation of many of these phthalate and non-phthalate compounds. For example, there is a lack of information regarding the toxicity of and the fate of the metabolites of alternative plasticizers (which is particularly important given that many of the negative health effects associated with DEHP are known to stem from its metabolites rather than the parent compound), including toxicological endpoints such as carcinogenicity and endocrine disruption [40]. As new concerns have been raised about some of these DEHP replacements, such as DINP [11,41,42,43,44,45], it is increasingly important to produce truly green replacement plasticizers, with the factors advancing hazard reduction playing a bigger role in plasticizer development. Furthermore, with hundreds of commercial plasticizers available today for numerous applications, it is important to ensure that these and new plasticizers are evaluated and designed systematically and thoroughly, to avoid the “regrettable substitution” of one problematic compound with another [35].

## 3. Designing Non-Toxic Chemicals

Given the large variety of plasticized PVC applications, including in sensitive materials such as hospital tubing, blood bags and children’s toys, ensuring the non-toxicity of green plasticizers is of utmost importance. Since several currently used phthalate plasticizers, such as DEHP and DINP, are suspected endocrine disruptors, particular attention should be paid to reproductive toxicity. This is, of course, no small task and collaboration between chemists and toxicologist can ensure that the challenge is met.

In the last decade, the availability of computing power to support complex tasks such as modelling the interactions of molecules with biological systems has increased, and, as a result, efforts are underway to use in silico (i.e., computational) methods to predict toxicity through, for example, quantitative structure-activity relationships (QSAR) [46]. These simulations are used to inform the earliest stage of chemical design, thereby helping to reduce the amount of costly experimental testing required of compound candidates [47,48,49]. In support of this goal, databases have been established that contain large inventories of chemical compounds and their known toxicological properties [24,47], which can, and should, act as important resources for designers of green plasticizers. While such approaches are very useful in the early stages of molecular design, toxicity tests in living systems will ultimately be required.

In accordance with the principles of green chemistry [24], toxicity considerations should influence plasticizer development as early as in the design phase of the molecule. To ensure that any designed green plasticizers are in fact non-toxic, it is imperative to measure different toxicity end points in a variety of species, which may also be required by regulators for new products entering the market. These tests range from bacterial assays and assays on mammalian cell lines to long-term in vivo studies. In the following sections, examples of safer chemical design and toxicity testing of plasticizers are presented. The list of examples is not intended to be exhaustive but is illustrative of such tests.

Bacterial assays have been used to estimate microbial plasticizer toxicity [50], however it is notable that most bacterial studies involving plasticizers have focused on the biodegradability of plasticizers after leaching from the resin [51,52,53,54,55,56]. In turn, this means that the acute microbial toxicity of plasticizers should not be overly concerning, since the bacteria are able to grow and feed on plasticizers as substrates in the biodegradation studies. Therefore, to address the question of reproductive toxicity, yeast-based assays have been developed for initial screening for estrogen agonists [57,58], but cell-based assays also exist [59]. In vitro assays using mammalian (or other) cell lines are regularly performed and these are used to asses a wide range of effects ranging from general toxicity (e.g., viability assays [60]), to whether cell growth and division is inhibited (proliferation assays), to gene expression, steroidogenesis, mitochondrial integrity, etc. [13,61,62,63,64]. It is particularly important to test the toxicity of plasticizer metabolites [63] since these can sometimes have greater adverse effects than their parent compounds [13,51]. Recent advances have also allowed for automated high-throughput screening (HTS) of chemicals, generating large in vitro databases such as ToxCast and Tox21 [65,66,67].

As a next step, in vivo studies are often performed. However, due to the labor-intensive and costly nature of in vivo experiments, only serious green plasticizer contenders should proceed to this testing stage. In vivo studies can test not only for general toxicity, but for more specific biological effects such as reproductive toxicity. This is done by conducting multi-generational experiments, examining both the parent animal and their offspring and monitoring various endpoints such as organ weight, steroid levels, sperm quality, and gene expression [45,68,69,70,71,72]. Numerous studies on DEHP and other phthalates demonstrate the reproductive effects of these compounds [45,68,69,70,71,72], yet in vivo studies for proposed alternative plasticizers are much less common. Some examples of such studies, mainly performed in rats, do exist, including the following:

- In a one-generational study, a “hyperbranched polyglycerol” plasticizer was shown not to be acutely toxic [65].
- In a two-generational study, two proposed green plasticizers, dioctyl succinate (DOS) and 1,4-butanediol dibenzoate (BDB), were both shown to exhibit no acute toxicity, and DOS also showed no reproductive toxicity, while BDB could produce “subtle but significant alterations of estrogen signaling in the adult testis” [34,66].
- In a two-generational study, commercially-available di(2-ethylhexyl) adipate (DEHA) was shown to have developmental toxicity at doses above 200 mg/kg/day as evidenced by increased postnatal deaths, yet no reproductive toxicity (antiandrogenic effects) was found [67].
- In several one- and two-generational studies, the commercially available DINCH (hydrogenated DINP) showed no acute toxic effect [68], yet there were some indications that it might have an effect on the developing reproductive system of male rats as well as a similar effect as observed with BDB (see above) [30,34,66].
- In a one-generational study, a plasticizer candidate closely resembling DINCH (“DL9TH”) was shown to be safe for adult rats, with a further claim that the compound also showed no reproductive toxicity. This was based on tests with adult animals, not a two-generational study [69].

## 4. Designing for Biodegradation

Toxicological risk is defined as a function of hazard and exposure [73]. The previous discussion on toxicity concerns the first term, hazard, which relates to the intrinsic chemical toxicity of the compound. While reducing or eliminating hazard is at the core of the twelve principles of green chemistry [24], a reduction in exposure would also lead to lower overall risk. Reducing exposure can be achieved by developing biodegradable compounds or by reducing migration and leaching of the plasticizer out of the polymer blend. Therefore, a truly green plasticizer would be a compound that would not be persistent in the environment nor produce stable or pseudo-persistent metabolites during its breakdown [23,74]. Pseudo-persistent compounds enter the environment (e.g., due to continuous plastic disposal) at a greater rate than they are removed. Monitoring the kinetics of degradation and, in particular, the fate of metabolites is a key component of plasticizer biodegradation testing, due to the known effects of plasticizer metabolites such as MEHP [13,14,16]. Thus, biodegradation is a crucial component of any assessment of green plasticizers.

However, assessing the biodegradation potential of a new or existing chemical is not always straightforward, since it can be influenced by many environmental factors including temperature, atmosphere (e.g., aerobic versus anaerobic), and the presence of specific soil and water microorganisms [75]. Furthermore, results can vary depending on the use of different test protocols. Several heuristics do exist and can be used as a starting point to design for degradability. For instance, ester groups, amides, oxygen in the form of hydroxyl, aldehyde, or carboxylic acid groups, unsubstituted linear alkyl chains, and phenyl rings are generally features that increase aerobic degradability. Conversely, strongly electron-withdrawing groups like chlorine, branched structures with a quaternary carbon, and highly substituted structures are less likely to be biodegradable [76]. As with any heuristic, exceptions to these rules can be found, however they are a useful starting point. A number of computer models also exist to predict the biodegradability of organic chemicals. Some commonly used models are Biowin, a group contribution model, and CATABOL, a knowledge-based system that can be used for predicting pathways [76].

Several varieties of tests exist to assess biodegradation experimentally. These include screening tests, simulation tests, and field tests. Screening tests are the simplest form of tests, where compounds are suspended in an aqueous solution, generally inoculated with a polyvalent inoculum (i.e., a mix of multiple microorganisms collected from local wastewater treatment plants, river water, soil, etc.). The most common screening tests are “ready” biodegradation tests and “inherent” biodegradation tests. Ready biodegradation tests provide a basic determination of whether a compound is “readily biodegradable” (and often result in an underestimation of biodegradation potential) while inherent biodegradation tests provide a fuller assessment of degradation potential by using higher inoculum concentrations, thereby creating a more favorable degradation environment [77]. Simulation tests are more sophisticated than screening tests and measure the rate and extent of biodegradation, usually in a continuous system designed to simulate real-life conditions such as anaerobic degradation occurring in a waste water treatment plant [78]. Field studies are the most complex, but least controlled, type of test which involve monitoring the degradation of the compound in a natural matrix [79]. The Organization for Economic Co-operation and Development (OECD) has defined several biodegradation tests (which fall under the categories of screening and simulation tests) based on measuring parameters such as oxygen consumption or carbon dioxide evolution as indicators of bacterial growth and compound mineralization (i.e., total breakdown of the compound to water and CO_2_). The OECD tests include closed bottle tests using sludge, obtained for example from wastewater treatment plants [78,80]. While these tests are rapid and easy to conduct, they possess some drawbacks, and suggestions for their improvement have been made [81]. Importantly, these tests, along with most other screening and simulation tests, do not call for metabolite analysis, possibly missing the presence of stable breakdown products that might go unnoticed following the standard protocol. As seen, this is particularly important when evaluating plasticizers, since commercial plasticizers, such as DEHP, have been shown to have stable metabolites (e.g., MEHP) that exhibit toxicity [13,14,16]. Since the task of monitoring metabolites can be complicated by the use activated sludge or other complex mixtures, biodegradation experiments using singe-strain cultures of common soil bacteria have also been developed, allowing for improved recovery of the hydrophobic plasticizer and metabolite molecules [51,52]. Of course, these experiments do not fully reflect degradation under natural conditions, however they can be particularly useful for comparisons between plasticizer groups, and for metabolite analyses [51,54].

**Figure 4:**
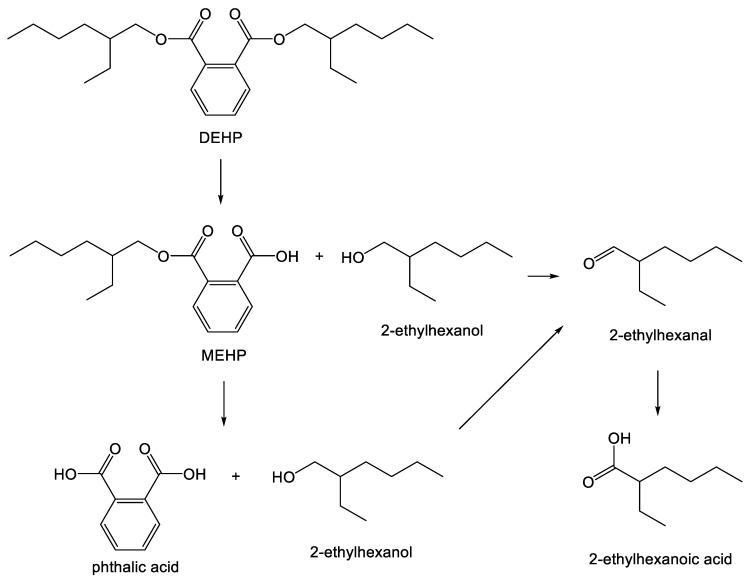
Biodegradation of di(2-ethylhexyl) phthalate (DEHP) through the action of esterases in microbes. Reprinted by permission from Springer Nature: Springer. Applied Microbiology and Biotechnology. Leaching of the plasticizer di(2-ethylhexyl)phthalate (DEHP) from plastic containers and the question of human exposure, Erythropel et. al., 2014 [4].

In order to design plasticizers for biodegradability, a common strategy is to examine the chemical structures of commercially-used plasticizers, seek to understand which functional groups cause slow biodegradation kinetics or toxicological implications, and then use this knowledge to re-design the molecule to circumvent these problematic properties, while ideally retaining plasticizing effectiveness. For example, the biodegradation of succinate, maleate, fumarate, adipate and dibenzoate diesters has previously been investigated [51,52,53,54,82,83,84,85,86,87]. In a first step to developing alternative biodegradable plasticizers, several common soil bacteria and yeasts were tested for their biodegradation potential, and *Rhodococcus rhodochrous* was identified as the most promising micro-organism to use in kinetic testing due to its ability to grow on hydrophobic substrates [51,52]. In the next step, the biodegradation pathway for DEHP was determined (see Figure 4). Briefly, DEHP biodegradation yields the following metabolites: MEHP, phthalic acid, and 2-ethyl hexanol, which is subsequently oxidized to 2-ethyl hexanoic acid [86,87,88]. Both MEHP and 2-ethylhexanoic acid have been shown to be persistent in the environment [89,90,91].

**Figure 5:**
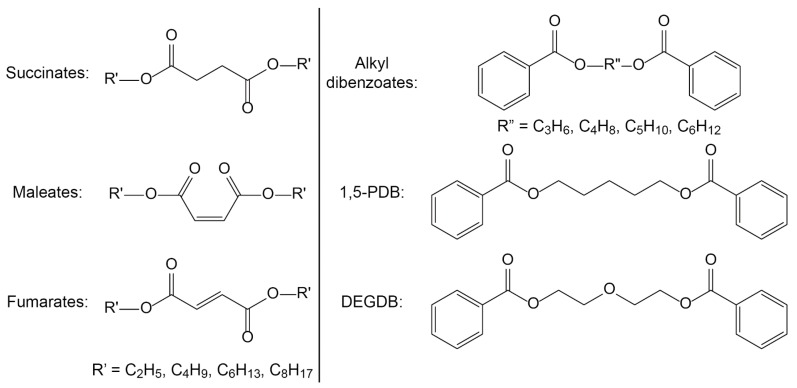
Candidate green plasticizer families: succinates, maleates, fumarates (all with unbranched side chains), and linear alkyl dibenzoates. The only difference between the dibenzoate 1,5-PDB and the commercial diethylene glycol dibenzoate (DEGDB) is the molecule in the center of the diol linker: carbon (in the case of 1,5-PDB) or oxygen (in the case of DEGDB). 1,5-PDB is 1,5-pentanediol dibenzoate, DEGDB is diethylene glycol dibenzoate.

Several candidate green plasticizers were designed based on these biodegradation pathways to avoid producing breakdown structures known to be toxic or persistent (see Figure 5). These included diesters based on succinic acid, maleic acid, and fumaric acid, which resemble the phthalate structure, esterified with linear alcohols to avoid the buildup of 2-ethylhexanoic acid following biodegradation of the parent compound [54,84,85,92]. The compounds were found to be effective plasticizers and biodegradation experiments revealed that the geometry of the central structure of the molecules played an important role in how quickly the compounds were degraded. The saturated succinate esters that can rotate around the central bond were more rapidly biodegraded by *R. rhodochrous* than the unsaturated maleates and fumarates (see Figure 5). Following similar steps, the dibenzoate plasticizer 1,5-propanediol dibenzoate (1,5-PDB) was designed with the intent that it would biodegrade much more quickly than the commercially-available diethylene glycol dibenzoate (DEGDB) by the simple replacement of an oxygen atom of the ether function in DEGDB with a carbon atom to form 1,5-PDB (see Figure 5). Both compounds also exhibited similar plasticizing effectiveness in PVC [93].

The biodegradability of candidate green plasticizers is still not a routine assessment. Consequently, few papers were found in the published literature on the topic. The focus of the limited number of papers that are available is often on the degradation behavior of the polymer blends (for example plasticizers blended with biodegradable polymers such as PHA or PLA) rather than the plasticizer itself. The lack of biodegradation work on plasticizers intended for use in PVC is a significant shortcoming since the development of biodegradable plasticizers could drastically reduce the environmental impact of this class of additives. A few examples of biodegradation studies for candidate plasticizers and their metabolites that can be used as guidelines for future testing include the following:

- Biodegradation testing of poly(caprolactone)-based plasticizers by *R. rhodochrous*, which demonstrated rapid biodegradability and no build-up of stable metabolites [92].
- Biodegradation testing of various dibenzoate plasticizers similar to 1,5-PDB, both in batch conditions and in a continuous bioreactor. While biodegradability was generally found to be good, the degradation of some compounds resulted in a build-up of toxic metabolites [44,80,81,91].
- Biodegradation testing of DEHP and 15 diesters of varying side chain length based on succinic acid, maleic acid, and fumaric acid by *R. rhodochrous*, as discussed above. The experiments revealed the influence of both central structure as well as side chain length and its branching on biodegradation kinetics [45,82,83].

## 5. Designing for Permanence

The concept of risk being a function of hazard and of exposure is also important when considering plasticizer leaching. Exposure can be reduced by increasing the permanence of plasticizers within blends, thereby limiting their leaching potential. This addresses the issue of acute exposure, for example from hospital tubing or blood bags, and has less bearing on chronic exposure since the plasticizer will still eventually leach from the blend, due to the fact that no chemical bond exists between the plasticizer and PVC. Even at very low leaching rates, plasticizers can eventually migrate from the blend into the environment, as observed in landfill sites or in natural environments where plastic waste is present over the long term [4,94]. Whether this leaching occurs over the timescale of months, years or decades, plasticizers will ultimately enter the environment and, if they are not readily biodegradable, their persistence and bioaccumulation (as seen for DEHP and other phthalates) will become an environmental problem [4,95]. Additionally, excessive leaching is also detrimental to the durability of the plastic product. The minimization of leaching is important both for the sake of the product performance and its safe use. Therefore, lowered or suppressed leaching rates are favourable to reduce acute human exposure, to minimize the scope of environmental contamination and to improve performance and are, therefore, important considerations in green plasticizer design.

Plasticizer leaching rates are closely tied to the compatibility and miscibility of the plasticizer within the PVC blend. Immiscible plasticizers will blend poorly with PVC and be at higher risk of leaching. Yet plasticizers that demonstrate good permanence often do not provide an adequate plasticization effect (see Figure 1). Striking a balance is important when designing a plasticizer with good plasticizer effectiveness, yet low rates of leaching. More detailed examination of the complex relationship between plasticizer compatibility and leaching rates is available [3,25,96]. Additionally, the molecular weight of plasticizers seems also to have an effect on leaching, as evidenced by decreased leaching rates into water with increasing molecular weight for several ester-based plasticizers [5,97]; however, this could also be related to the low water solubilities of these higher molecular weight plasticizers.

Given this complexity, the experimental determination of plasticizer leaching rates is required to ensure both good plasticizing performance and low acute exposure. ASTM D-1239 outlines a standard testing method [98] to test for leaching into a variety of matrices including water, soapy water (1% soap), cottonseed oil, mineral oil, kerosene, and ethanol (50% in water) to accommodate for a variety of environments into which leaching can occur. Leaching rates of plasticizers into aqueous media is of particular importance since this is the most representative of actual plasticizer leaching into the environment, and many studies of proposed alternative plasticizers have focused on this. While not an exhaustive list, examples of leaching studies include the following:

- Leaching of the commercial plasticizers DEHP, DINCH, TOTM/TEHTM and di(2-ehtlyhexyl) terephthalate (DEHT) from hospital tubing into 50% ethanol in water [99].
- Leaching of several commercial plasticizers including phthalates and DEHA found in food packaging into aqueous acetic acid (3%), distilled water and ethanol (15% in water) [100].
- Leaching of alternative dibenzoate, succinate, maleate, and fumarate-based plasticizers from PVC disks at 29 wt % loading into reverse-osmosis purified water [4].
- Leaching of oligomeric ɛ-caprolactone in PVC disks at 39 wt % loading in *n*-hexane [92].
- Leaching of oligomeric poly(butylene adipate) in PVC films at 40 wt % loading into water [97].
- Leaching of curcumin-derived plasticizer candidates at 5, 15, 25, and 35 wt % in PVC into water and *n*-hexane [101].
- Leaching of tetra-esters based on pentaerythritol at several concentrations in PVC into distilled water, olive oil, ethanol (10% in water), acetic acid (30% in water), and petroleum ether [102].
- Leaching of DEHP from hemodialysis tubing, with and without polyurethane coating, into newborn calf serum [103].

Several techniques have been explored to avoid leaching, including internal plasticization [99,100,101], coating of polymer surfaces [102], and plasma surface treatment [103,104] to create a barrier through which plasticizer molecules cannot penetrate. Most of these techniques require further processing of the plasticized material, thereby making the product more expensive, more complicated to produce, and sometimes resulting in a decrease in plasticizer effectiveness [105].

## 6. Green Production

While this review has focused largely on the experimental assessment and design of green plasticizers, the chemical synthesis and scale-up of the production of these compounds should also be considered, though there is a lack of literature in this area. In order for a plasticizer to be deemed green, it is not sufficient to examine only the hazards associated with the compound itself, rather one must also consider how the compound is produced, including feedstock sourcing and synthesis methods.

Once a candidate plasticizer has been assessed and deemed suitable in terms of its performance, toxicity, biodegradation and leaching, it is important to scrutinize the synthetic techniques that are employed in its production using green chemistry principles. A number of these principles concern chemical synthesis and can be applied to plasticizers such as the use of safer solvents and auxiliaries, less hazardous chemical synthesis, waste prevention, atom economy, catalysis, and reduced number of derivatives, but also energy efficiency and real-time analysis for pollution prevention [23]. However, there is a lack of published work on the topic of green synthetic techniques applied specifically to plasticizer production. Nevertheless, the aforementioned principles can be applied as a starting point for new research.

The use of renewable feedstocks for plasticizer production rather than the use of petroleum-based feedstocks should be considered when designing a truly green plasticizer. The most commonly used class of plasticizers are esters, which are made up of organic acids esterified with alcohols, of which there is increasing renewable supply available. For example, a report by the U.S. Department of Energy Biomass program identified a range of “building block” compounds including small organic acids and alcohols, that are accessible from renewable sources [106]. Starting materials include starches, sugars, and wood components such as cellulose, hemicellulose, and lignin, as well as oils and proteins [106]. One compound that has garnered considerable interest as a renewable feedstock is succinic acid, which is already produced by fermentation at an industrial scale and can be used as a good platform chemical as-is, or by its further reduction to 1,4-butanediol [107,108]. Plasticizers based on succinic acid have been explored in several recent studies [85,109].

Special consideration should be given to using renewable materials that do not displace food production in order to avoid important social and economic repercussions [110]. For instance, renewable materials that are derived from agro-industrial residues and from non-edible biomass can be used as chemical feedstock for plasticizer synthesis [111]. An analysis of renewably sourced materials by LCA procedures is generally recommended.

It is worth noting that one of the key limitations of this discussion is that it has focused exclusively only on plasticizer hazards, and begs the follow-up question “how green is your product?”. Since most plasticizers are used with PVC, a non-renewable and petroleum-sourced polymer, there is much work that still needs to be done in the realm of improving vinyl chemistry to make it more sustainable.

## 7. Conclusions

Considerable care should be taken when designing green plasticizers. The “design” stage itself cannot be stressed enough as it will have a huge bearing on the properties of the final product, and only a well-designed plasticizer can strive to meet the highest standards that are needed for wide-ranging, and often sensitive, PVC applications. The designer of a green plasticizer should be concerned not only with the effectiveness of the compound in plasticizing PVC, although this remains an indisputable prerequisite, but also with its behavior once in contact the human body and the environment, throughout its entire life cycle. While the term “green” is often used loosely to characterize compounds that have been improved with respect to one, or a few, of the criteria discussed in this review, in order for a compound to be truly green it must be evaluated broadly against many different principles. In the case of green plasticizers for PVC, we suggest endeavoring to create compounds that do not possess negative health consequences, form harmful metabolites, or persist in the environment. Beyond these three key parameters, improving synthetic steps in accordance with the principles of green chemistry and utilizing renewable feedstocks when available is also important. As a guiding framework, the concepts of green chemistry are very suitable for confronting the task at hand. It is particularly important to integrate knowledge and expertise from different disciplines in order to address the complex and varied concerns of green design. In order to not repeat the mistakes of the past, it is crucial for any plasticizer designer to address these issues together and often in parallel, rather than separately, for only in this way can a genuinely safe, and thereby green, plasticizer be designed in an efficient manner.
